# Patient understanding of radiation risk from medical computed tomography—A comparison of Hispanic vs. non-Hispanic emergency department populations

**DOI:** 10.7717/peerj.937

**Published:** 2015-05-07

**Authors:** Afton McNierney-Moore, Cynthia Smith, Jose Guardiola, K. Tom Xu, Peter B. Richman

**Affiliations:** 1 Department of Emergency Medicine, Christus Spohn/Texas A&M School of Medicine, Corpus Christi, TX, USA; 2 Texas A&M University/Corpus Christi, Corpus Christi, TX, USA; 3 Department of Surgery, Texas Tech University Health Sciences Center, Lubbock, TX, USA

**Keywords:** Radiation risk, CT, Patient knowledge

## Abstract

**Background.** Cultural differences and language barriers may adversely impact patients with respect to understanding the risks/benefits of medical testing.

**Objective.** We hypothesized that there would be no difference in Hispanic vs. non-Hispanic patients’ knowledge of radiation risk that results from CT of the abdomen/pelvis (CTAP).

**Methods.** We enrolled a convenience sample of adults at an inner-city emergency department (ED). Patients provided written answers to rate agreement on a 10-point scale for two correct statements comparing radiation exposure equality between: CTAP and 5 years of background radiation (question 1); CTAP and 200 chest x-rays (question 3). Patients also rated their agreement that multiple CT scans increase the lifetime cancer risk (question 2). Scores of >8 were considered good knowledge. Multivariate logistic regression analyses were performed to estimate the independent effect of the Hispanic variable.

**Results.** 600 patients in the study group; 63% Hispanic, mean age 39.2 ± 13.9 years. Hispanics and non-Hispanics whites were similar with respect to good knowledge-level answers to question 1 (17.3 vs. 15.1%; OR = 1.2; 95% CI [0.74–2.0]), question 2 (31.2 vs. 39.3%; OR = 0.76; 95% CI [0.54–1.1]), and question 3 (15.2 vs. 16.5%; OR = 1.1; 95% CI [0.66–1.8]). Compared to patients who earned <20,000, patients with income >40,000 were more likely to answer question 2 with good knowledge (OR = 1.96; 95% CI [1.2–3.1]).

**Conclusion.** The study group’s overall knowledge of radiation risk was poor, but we did not find significant differences between Hispanic vs. non-Hispanic patients.

## Introduction

Considering the impact of radiation exposure on lifetime malignancy risk, regulators are increasingly scrutinizing the utilization of computed tomography (CT) (Smith-Bindman et al., 2012; Medicare Improvements for Patients and Providers Act of 2008, 2008; Berrington de González et al., 2009; Smith-Bindman et al., 2009). In 2008, the Medicare Improvements for Patients and Providers Act established conditions of participation for imaging facilities. Among the many conditions required for Medicare reimbursement eligibility are appropriateness criteria for imaging and radiation protection guidelines (Medicare Improvements for Patients and Providers Act of 2008). Thus, hospitals face risk for lower revenues if administrators fail to establish polices and procedures for safe utilization of modalities that expose patients to radiation.

The emergency department (ED) represents an obvious point of focus for radiation risk reduction interventions in view of the high volume of radiological procedures that originate in this area of the hospital. Prior investigative work has revealed that emergency department patients, physicians, and even radiologists typically underestimate the potential harm associated with CT (Baumann et al., 2011; Lee et al., 2004; Takakuwa, Estepa & Shofer, 2010; Krille et al., 2010; Caoili et al., 2009; Arslanoglu et al., 2007; Ludwig & Turner, 2002; Liberman & Eddy, 1995; Zwank, Leow & Anderson, 2014). However, there is limited data to describe how cultural and ethnic influences may impact comprehension of such risks. Factors such as language barriers for available news or educational materials may potentially render certain populations less familiar with this subject matter. Latinos may be a particularly vulnerable population from this perspective, and the need for further focus on this group in medical research is reflected by the fact that Hispanics have accounted for over half of the overall US population growth during a decade period (The Hispanic Population in the United States, 2010).

Although Takakuwa, Estepa & Shofer (2010) previously noted that non-Caucasians, as compared to Caucasians, had lower levels of knowledge regarding risks from radiological studies, this population had a very low proportion of Hispanic patients. The purpose of our current study was to determine whether this growing subset of the population might require specific intervention to educate them further on this key public health concern. Specifically, we conducted a survey to assess differences in Hispanic versus non-Hispanic patients’ knowledge of radiation risk in our inner city academic emergency department with respect to their understanding of relative radiation exposure. The study was designed to test the null hypothesis that there would be no difference in level of knowledge regarding radiation risk from a CT of the abdomen/pelvis between Hispanic and non-Hispanic patients.

## Methods

Study Design–This was a cross-sectional, prospective study designed to evaluate the knowledge of patients with respect to radiation risk from medical imaging.

Setting–The study was conducted at Christus Spohn Memorial Hospital (Corpus Christi, TX). The facility is a major teaching affiliate of Texas A&M Health Science Center, a level-two trauma center, and serves an inner-city population with an annual Emergency Department (ED) census of 45,000 patients. The Christus Spohn Institutional Review Board approved the study prior to the initiation of data collection (study #13–021). Verbal consent was provided by study participants at point of enrollment.

Population–Our study included a convenience sample of medically stable, consenting, adult patients age >18 years that presented to the emergency department. Patients were excluded for any of the following reasons: refusal to provide consent, pregnancy, and inability to complete the questionnaire due to clinical instability, severe pain, or disorientation as determined by a study physician. Patients that were not English or Spanish speaking were also excluded as our written study materials were only available in those languages.

Study Protocol–Consecutive, consenting eligible patients were enrolled during a 6-week period (November/December 2013) during hours at which trained research associates were available to assist with data collection. The knowledge assessment instrument represents a modification of the methods of Takakuwa, Estepa & Shofer (2010) to assess patient knowledge of radiation risk through a series of factual questions comparing radiation exposure from CT to other forms of exposure (e.g., plain film radiography). In a similar fashion, patients provided written answers to collect basic demographic information including sex, age, race, income, and education as well as answers to the primary study questions. The knowledge assessment tool then asked participants three questions designed to measure their knowledge of radiation exposure from a CT scan of the abdomen/pelvis (CTAP). Patients were asked to rate their level of agreement on a 10-point scale for two factually correct statements comparing radiation exposure equality between: CTAP and 5 years of background radiation exposure (question 1); CTAP and 200 plain film chest x-rays (question 3). Patients were also asked about their level of agreement that multiple CT scans increase lifetime risk of cancer (question 2).

Statistical Analysis–Data was entered into Excel for Windows (Microsoft Corporation, Redmund, Washington, USA) and transported into STATA software (STATA, College Station, Texas, USA) for analysis. Descriptive statistics of all variables are first provided. During study design, authors/study statistician decided that patient responses to the three questions would be dichotimized to scoring 8 or higher (good knowledge) vs. 7 or lower. Age (65 or older vs. younger) and the highest educational achievement (high school graduate vs. no high school diploma) were used as binary variables. The race and ethnicity variable was grouped into non-Hispanic white (NHW), Hispanic, and other races/ethnicities. Annual income level was categorized as $20,000 or lower, $20,001-40,000 and greater than $40,000. Bivariate analyses were then performed on whether Hispanic ethnicity was associated the ratings of the three questions. To control for confounding, multivariate logistic regression analyses were performed to estimate the independent effect of the Hispanic variable. Odds ratios and 95% confidence intervals were calculated. Alpha was set at 0.05. The primary outcome parameter was to compare the percentage of Hispanic vs. non-Hispanic patients who revealed “good” knowledge to each question with the goal of testing the null hypothesis that there would be no significant differences between the groups.

## Results

We enrolled 600 patients who successfully completed (Table 1). Within the study group, 62.5% were Hispanic and just over half were female (53.5%). The mean age was 39.2 ± 13.9 years. The study population came from a predominantly lower socioeconomic status and was poorly educated. Patients reported an annual income <$40,000 in 85% of cases. Meanwhile, 29% reported less than a high school education.

**Table 1:** Descriptive statistics (%).

		Question 1 score ≥8 (16.33%)	Question 2 score ≥8 (33.33%)	Question 3 score ≥8 (15.50%)
Age				
<65	94.50	16.23	33.33	**14.81**
65 +	5.50	18.18	33.33	**27.27**
Gender				
Male	46.48	16.61	34.66	14.08
Female	53.52	16.30	32.60	16.61
Race/Ethnicity				
Non-Hispanic White (NHW)	29.67	15.17	39.33	15.17
Hispanic	62.50	17.33	31.20	16.53
Other races	7.83	12.77	27.66	8.51
Annual income				
≤$20,000	60.83	15.89	**27.67**	16.16
$20,000–40,000	22.83	14.60	**41.61**	13.87
>$40,000	16.33	20.41	**42.86**	15.31
Education				
High school graduates	41.83	16.33	34.26	15.54
No high school diploma	58.17	16.33	32.66	15.47

Table 2 summarizes the results of multivariate logistic regression. Hispanics and non-Hispanic whites were similar with respect to good knowledge-level answers to question 1 (CT abdomen/pelvis vs. 5 years background radiation exposure; 17.3 vs 15.1%; OR = 1.2; 95 % CI [0.74–2.0]), question 2 (lifetime risk of cancer following CT exposure; 31.2 vs. 39.3%; OR = 0.76; 95% CI [0.54–1.1]) and question 3 (CT abdomen/pelvis vs. 200 plain film chest-xray; 15.2 vs. 16.5%; OR = 1.1; 95% CI [0.66–1.8]).

**Table 2:** Multivariate logistic regression.

	Question 1 Score ≥8		Question 2 Score ≥8		Question 3 Score ≥8	
	OR	*p*	OR	*p*	OR	*p*
Age 65+ (vs. <65)	1.09	0.855	0.95	0.895	2.04	0.084
Female (vs. male)	0.98	0.929	0.96	0.803	1.17	0.502
Race/Ethnicity (vs. NHW)						
Hispanic	1.21	0.454	0.76	0.158	1.08	0.751
Others	0.86	0.764	0.63	0.207	0.57	0.315
Income (vs. ≤$20,000)						
$20,000–40,000	0.93	0.789	**1.82**	0.004	0.82	0.493
>$40,000	1.41	0.243	**1.96**	0.005	0.95	0.867
<High school (vs. ≥ high school)	0.97	0.907	0.95	0.786	0.97	0.907

There was no significant association between the following variables and good knowledge-level answers to questions 1, 2, or 3, respectively: education, gender, age. However, as compared to patients who earned <$20,000, patients within higher income segments were more likely to provide good knowledge-level answers to question 2, including $20,000-$40,000 (OR = 1.8; 95% CI [1.2–2.8]) and >$40,000 (OR = 1.96; 95% CI [1.2–3.1]).

## Discussion

The use of medical computed tomography (CT) has expanded dramatically in recent years with a resultant increase in the emergency physician’s certainty of diagnosis as well as a reduction in the need for emergency surgery (Rosen et al., 2000; Rosen et al., 2003). The improving speed of modern CT scanners has made it an increasingly useful diagnostic tool in the high volume ED setting. According to appropriateness criteria established by the American College of Radiology, CT is currently the radiological study of choice for emergent evaluation of numerous symptoms, including acute flank pain and new onset headache (American College of Radiology Appropriateness Criteria, 2012).

In view of the diagnostic benefits of this imaging modality, it is not surprising that CT use has grown in a non-linear fashion over the past 15 years. Investigators reviewing the National Hospital Ambulatory Medical Care Survey observed that utilization of CT grew more than 10 times faster than the rate of emergency department visits from 1996 through 2007. In 1996, approximately 3.2 percent of emergency patients received a CT scan. By 2007, that number had risen to almost 14 percent (Koc Faze, Krumholz & Nallamothu, 2011). Similarly, in a study examining the medical records of over 1 million patients within a large integrated health care system from Midwestern states during the period 1996–2010, Smith-Bindman et al. (2012) reported that radiation exposure doubled. By 2010, for every 100 adult patients, 20 CTs were performed. Older patients underwent even more CT scans. For every 100 patients age 65 to 75, approximately 35 CTs were obtained. The financial burden to the Medicare system alone for high tech radiological scans for the elderly has grown to more than $14B annually (Medicare Improvements for Patients and Providers Act of 2008, 2008).

The increased use of CT over the past decade has significant potential risks to patients and the healthcare system beyond incurred costs. Unfortunately, there is a paucity of longitudinal data involving adult patients with exposure to CT imaging (Shuryak, Lubin & Brenner, 2014). Thus, estimates of radiation risk have been typically been extrapolated from pediatric studies or estimated from analogous human exposure doses from nuclear explosions that some authors caution may be imprecise (Canadian Agency for Drugs and Technologies in Health, 2014; National Research Council, 2014).

With those caveats in mind, there remains a general concern in the literature that CT utilization may lead to future deaths from exposure to the imaging modality. Berrington de González et al. (2009) estimated that approximately 29,000 future cancers could be related to CT scans performed in the U.S. in 2007 alone. Similarly, Smith-Bindham projected that 1 in 270 women and 1 in 600 men who undergo CT coronary angiography at age 40 will develop cancer from that CT scan; the risk for 20-year-olds are estimated to be roughly twice as large, and those for 60-year-olds are estimated to be roughly half as large (Smith-Bindman et al., 2009). Further, while noting the risk of mortality from acute injuries outweighed longer-term radiation concerns, Laack et al. (2011) estimated the risk of cancer death from CT as approximately 1 in 1,000 for trauma patients.

Considering the potential long-term risks, it is surprising that physicians, patients and even radiologists typically underestimate the potential harm associated from CT (Baumann et al., 2011; Lee et al., 2004; Takakuwa, Estepa & Shofer, 2010; Krille et al., 2010; Caoili et al., 2009; Arslanoglu et al., 2007; Ludwig & Turner, 2002; Liberman & Eddy, 1995; Zwank, Leow & Anderson, 2014).

Patient’s often rely on the ordering physician to educate them about the risks and benefits of medical imaging; however, the physicians themselves may be unaware of the radiation risks associated with such studies. In a large systematic review of studies assessing physician’s knowledge about radiation dose and medical imaging using 8 databases, physicians were found to have significant knowledge gaps in terms of medical risks associated with CT imaging (Krille et al., 2010). This was demonstrated in a study by Arslanogui et al. when 93.1% of surveyed doctors underestimated radiation dosing with medical imaging (Arslanoglu et al., 2007). Lee et al. (2004) found similar results when both Emergency Department physicians and Radiologists were surveyed and only 9% and 47% of those physicians reported increased risk of cancer associated with CT scans respectfully. Such findings have also been observed in medical training programs at a point in which there is a chance to better educate our future physicians. Sadigh et al. (2014) surveyed residents from 15 specialties at a major medical center and found that knowledge of radiation risk was “limited” despite regular emphasis on this topic within program curriculums.

There is an increasing trend towards including patients in the medical decision making process. However, a majority of patients are not aware of the risks associated with radiologic imaging. In a study done by Caoili et al. (2009), only 6% of patients knew that CT radiation increased the lifetime risk of cancer. This was similar to the findings done by Lee et at. demonstrating only 3% of surveyed Emergency Department patients believed CT scans were associated with an increased risk of cancer. Lee et al. (2004) Concordantly, when patients were asked to compare radiation dose of CT versus plain chest radiography, 70% of patients underestimated the dose. Those same patients, again, demonstrated poor comprehension of radiation and cancer risk. Unfortunately, they also reported increased confidence in their medical evaluation when CT imaging was done (Baumann et al., 2011). More recent studies suggest that patient knowledge of potential cancer risks from medical radiation exposure has significantly improved over the past decade, but the majority of patients continue to have limited awareness (Zwank, Leow & Anderson, 2014).

We believe a key question for researchers in this area is to identify whether patients within different demographic, educational, ethnic, and racial backgrounds might have different levels of knowledge about potential harm from imaging. This will allow educators to identify where the gaps of knowledge are and target them appropriately. Factors such as language barriers for available reading or educational materials may render certain populations less familiar with this subject matter. Latinos may be a particularly vulnerable population from this perspective, and the need for further focus on this group in medical research is reflected by the fact that Hispanics have accounted for over half of the overall US population growth during a decade period (Zwank, Leow & Anderson, 2014).

Takakuwa, Estepa & Shofer (2010) demonstrated that non-Caucasians as compared to Caucasians had lower levels of knowledge regarding risks from radiological studies. That survey reported 52% white, 40% black and 8% other race demographics, therefore we conducted our survey to better assess the knowledge base in the Hispanic population as this was poorly represented in the current data. Surprisingly, we did not find a significant difference in Hispanic versus non-Hispanic knowledge regarding radiation dose of CT abdomen and pelvis versus background radiation or plain chest radiography. We also did not demonstrate a significant difference in Hispanic versus non-Hispanic knowledge in regards to CT imaging and increased lifetime risk of cancer. However, our data set did mirror previous data demonstrating poor overall knowledge amongst emergency department patients about radiation dosing and cancer risk, particularly in the poorly educated and lower socio-economic populations.

## Limitations and future questions

This study has several limitations that warrant discussion. First, the study did not represent a true consecutive sample. Patients were enrolled consecutively during hours at which trained research associates were available. As the hours of the research associates varied throughout the hours of the day and week, we are hopeful that we surveyed a representative sample of our ED census including working and non-working patients.

Our results may also only be applicable in similar populations where the socioeconomic status is relatively low and with a predominance of Hispanics within the population. Anecdotally, we have a rather high percentage of Hispanic patients with fluency in English relative to other centers that our authors have worked in the Southwest. Thus, we may have accepted the null hypothesis but in a geographic area with a smaller percentage of English speaking Hispanics, we might have observed a different result.

Another limitation of the study is the potential for different methods of assessment and measurement to have provided a different result. We chose to use radiation dose from abdomen/pelvis CT as the CT reference comparison in the knowledge assessment tool due to its relatively high exposure risk. It is unclear that if we utilized CT of other body areas (e.g., thorax) and/or other types of radiation exposure for the non-CT reference whether our findings would have been similar. Likewise, the cut-offs for the level of understanding on the numerical scale are somewhat arbitrary and have not been validated elsewhere. We attempted to minimize this problem by examining the patients’ answers in several ways, including defining perfect knowledge as full agreement on the scale.

We note that CT imaging is ever advancing in quality and safety. Emergency physicians are increasingly aware and willing to discuss the topic at point of care (Griffey, Jeffe & Bailey, 2014). However, the appropriate information regarding degrees of radiation exposure is a “moving target” as they try to educate their patients over time. Patient radiation dose from the same type of scan has been rapidly decreasing through several changes in approach. Research has shown that simply engaging CT technologists with feedback from dose audits can lead to profound reductions in radiation exposure for subsequent patients (Miglioretti et al., 2014). New techniques to moderate exposure to areas of different density on the same imaging study, a process known as automated exposure control, can further reduce doses by 40%–70% alone (Hricak et al., 2011; Funama et al., 2012). In addition, new iterative software techniques are markedly reducing exposure in an additive fashion to those previously mentioned (Canadian Agency for Drugs and Technologies in Health, 2014). Such advances are ever critical when patients are showing an increased preference for CT alternatives to more invasive techniques such as coronary angiography and colonosopcy (Schonenberger et al., 2007; Svensson et al., 2002; La Grutta et al., 2014).

## Conclusions

Although the study group’s overall knowledge of radiation risk was poor, we did not find significant differences for Hispanic vs. non-Hispanic patients. Given the known risk of malignancy with exposure to radiation, it is our duty as physicians to inform our patients about the risks of undergoing CT evaluation. Our results suggest that physicians should assume poor knowledge and a need to counsel patients in lower socioeconomic groups about radiation exposure/diagnostic benefits of CT imaging.

.

## Supplemental Information

10.7717/peerj.937/supp-1 Appendix S1 Survey instrument

10.7717/peerj.937/supp-2 Supplemental Information 2 Radiation knowledge data file Radiation knowledge data.

10.7717/peerj.937/supp-3 Supplemental Information 3 Data collection sheet with verbal patient consent script and documentation Data collection sheet with verbal patient consent script and documentation
