# Supplementary material for: Patient understanding of radiation risk from medical computed tomography—A comparison of Hispanic vs. non-Hispanic emergency department populations
Source: PeerJ. 2015 May 7;3:e937. doi: 10.7717/peerj.937 (PMC4435503; doi:10.7717/peerj.937)
Supplement: Appendix S1 [file peerj-03-937-s001.pdf]

APPENDIX 1.

Radiation Risk Study

We are conducting a research study to test your level of knowledge of radiation risk/exposure. For that purpose, we have created a written survey for which we will ask you to answer a series of questions about your background and what you perceive your radiation risk to be from having CT scans. This survey will take approximately 5 minutes of your time.

Your participation in this survey is completely voluntary and will have no effect on your relationship with CHRISTUS Spohn Hospital Corpus Christi--Memorial as a patient. There will be no financial benefits for participating in this survey. Please know that I will do everything I can to protect your privacy. I will not identify you and all information that is recorded will be stored in a secure location.

Do you agree to voluntarily be a subject of this research study? ______**YES** ______**NO**

1. Sex: Male[1] Female[2]

2. Age in years: _________ **(If over 85 check here only)**

3. Ethnicity/Race: Caucasion/White[1] Hispanic[2] Native American[3] Asian[4] Non-Hispanic/Black[5] Other[6]

4. What is your household's yearly annual income (estimate)? (PLEASE CIRCLE ONE)

   \$0-\$20,000[1] \$20,001-\$40,000[2] \$40,001-\$60,000[3] \$60,001-\$80,000[4] \$80,001 or more[5]

5. What is the highest level of education you completed in school?

   Less than High School[1] High School Graduate[2] Vocational/Tech School Graduate[3] Some College[4] College Graduate[5] Any Post Graduate Work[6]

How much do you agree with the following statements?

Please use a scale of 0-10 **(PLEASE CIRCLE ONE)**

0= I Don't Understand the Question 1= Completely Disagree 5= Neither agree or disagree 10 = Completely Agree

6. A single CT scan of the Abdomen/Pelvis is the same as the amount of radiation the average person gets in 5 years from the sun/atmosphere.

   0 1 2 3 4 5 6 7 8 9 10

7. Having 7 CT scans of the Abdomen/Pelvis over a lifetime will increase your risk of cancer.

   0 1 2 3 4 5 6 7 8 9 10

8. A single CT scan of the Abdomen/Pelvis is the same as the radiation dose of over 200 single view chest x-rays.

   0 1 2 3 4 5 6 7 8 9 10
