# Supplementary material for: Patient understanding of radiation risk from medical computed tomography—A comparison of Hispanic vs. non-Hispanic emergency department populations
Source: PeerJ. 2015 May 7;3:e937. doi: 10.7717/peerj.937 (PMC4435503; doi:10.7717/peerj.937)
Supplement: Supplemental Information 3 — Data collection sheet with verbal patient consent script and documentation [file peerj-03-937-s003.docx]

**Radiation Risk Study**

We are conducting a research study to test your level of knowledge of radiation risk/exposure. For that purpose, we have created a written survey for which we will ask you to answer a series of questions about your background and what you perceive your radiation risk to be from having CT scans. This survey will take approximately 5 minutes of your time.

Your participation in this survey is completely voluntary and will have no effect on your relationship with CHRISTUS Spohn Hospital Corpus Christi--Memorial as a patient. There will be no financial benefits for participating in this survey. Please know that I will do everything I can to protect your privacy. I will not identify you and all information that is recorded will be stored in a secure location.

Do you agree to voluntarily be a subject of this research study? **______YES ______NO**

1. Sex: Male^1^ Female^2^
2. Age in years: ___________ **(If over 85 check here only)**
3. Ethnicity/Race: Caucasion/White^1^ Hispanic^2^ Native American^3^ Asian^4^ Non-Hispanic/Black^5^ Other^6^
4. What is your household’s yearly annual income (estimate)?  (PLEASE CIRCLE ONE)

          $0-$20,000^1^       $20,001-$40,000^2^     $40,001-$60,000^3^     $60,001-$80,000^4^     $80,001 or more^5^

1. What is the highest level of education you completed in school?

Less than High School^1^       High School Graduate^2^      Vocational/Tech School Graduate^3^

Some College^4^    College Graduate^5^      Any Post Graduate Work^6^

**How much do you agree with the following statements?**

Please use a scale of 0-10  **(PLEASE CIRCLE ONE)**

**0= I Don’t Understand the Question 1= Completely Disagree    5= Neither agree or disagree    10 = Completely Agree**

1. A single CT scan of the Abdomen/Pelvis is the same as the amount of radiation the average person gets in 5 years from the sun/atmosphere.
2. **1 2 3 4 5         6 7 8 9 10**
3. Having 7 CT scans of the Abdomen/Pelvis over a lifetime will increase your risk of cancer.
4. **1 2 3 4 5         6 7 8 9 10**
5. A single CT scan of the Abdomen/Pelvis is the same as the radiation dose of over 200 single view chest x-rays.

**0 1 2 3 4 5         6 7 8 9 10**
